# On the Recent Advances in the Theory of Vision

**Published:** 1872-09

**Authors:** H. Helmholtz, F. W. Abbott

**Affiliations:** The Royal Society of London


### Translations.

### *On the recent Advances in the Theory of Vision. By* H. HELMHOLTZ, *(of the Royal Society of London.)*

Translated from *Les Annales d' Oculistique,* By F. W. ABBOTT, M. D.

*Continued.*

In connection with the subject of these experiments upon the antagonism of the colors, a dispute has arisen between some excellent observers, a characteristic circumstance as it regards the nature of the phenomena. Some, among whom are found Dove, Regnault, Bruecke, Ludwig, Panum and Hering, affirm that they have seen the resulting color at the time of the binocular combination of the two colors. Others, such as H. Meyer (of Zurich,) Volkmann, Messner and Funke, declare just as positively that they have never seen the resulting color. For my part I do not hesitate to adopt the opinion of the latter, for attentive study of the cases in which I might have thought that I saw the resulting color has demonstrated to me that I had to do with phenomena of contrast. It was sufficient every time to look at the real resulting color by the side of the binocular mixture of the colors, to determine that it differed sharply from that of the mixture. It is nevertheless beyond a doubt that the first named observers saw what they said they did, and consequently that great individual differences exist here. In certain cases noted by Dove as particularly adopted to confirm his opinion, such as the binocular fusion of the complementary colors of polarization so as to form white, I have never been able to determine the existence of the least trace of a mixture.

This remarkable divergence upon the subject of an observation relatively so simple has appeared to me the more interesting, since it comes to the support of this hypothesis of the empirical theory, according to which, in general, we consider as separated in space only those sensations whose separation we can obtain by the aid of voluntary movements. According to the theory of Young, when we look with one eye alone at a mixed color, there is still formed three different sensations; but no movement of the eyes can separate them, they always remain united locally. Nevertheless we have seen that exceptionally a different localization in the notions of these colors is still formed, as soon as a part of the color seems to appertain to a transparent covering. When two corresponding points of the retinas are illuminated by different colors, the separation of these colors does not often occur in ordinary vision, and when it does take place, it is generally in the parts of the visual field to which we are not giving attention. The germ of such a separation into two parts which, during the movement of the eyes, move up to a certain point of independently of each other, exists none the less; according to the degree of attention which the observer is accustomed to accord to the peripheral parts of the field of vision and to double images, he will become more or less capable of isolating the colors which strike both retinas simultaneously. Mixtures of colors, monocular or binocular, simultaneously excite several sensations of color whose localization is the same in the field of vision. The difference between the notions obtained consists in this, that sometimes we interpret this system of sensations as being an indivisible whole, and that sometimes previous exercise enables us to analyze it into its constituent parts. We almost invariably proceed in the first way before a monocular mixture of colors, while we are more likely to proceed in the second manner when we have to do with a binocular mixture. But as this tendency is necessarily founded upon an acquired habit, it is easy to understand that it may present great individual variations.

If we observe the antagonism which is produced by the combination of two stereoscopic designs, one of which is executed in black upon a white ground, the other in white on a black ground, we remark that the black and white lines nearly corresponding always remain visible one beside another, which can only obtain if both back grounds, white and black, persist simultaneously. Then, upon a back ground with a lustre like that of graphite, we see an impression produced much more calm than would be produced by the antagonism of two entirely different designs. The experiment becomes more striking by adding a white printed leaf by the side of the part of the design which is upon a black ground, so that the black ground exhibits during binocular vision upon one side the effect of luster, and on the other that of antagonism. So long as we direct our attention to the form of the represented object, so long as we survey it with our eyes, the differently colored contours concurrently command the movement of the eyes, and fixation can only be maintained by the *simultaneous* fixation of two lines. This demands that the attention should be directed all the time to both designs, and it is clear that both impressions persist then with the same vividness. There does not exist a better means of permanently preserving the simultaneous impression of both images. It is possible, to be sure, to see in places, for a little time, the combination obtained by the super position of two dis similar designs; we may, to aid ourselves, observe the manner in which they cover each other, the angles at which the lines of which they are formed intersect &c. But when we fix the sight upon one of these lines the field which does not contain it immediately disappears.

Let us recapitulate the facts relating to binocular vision.

1st. Irritations of corresponding points of the two retinas do not fuse into an indecomposable impression, because, if it were so, we could not conceive of the production of stereoscopic luster. We have seen above that this phenomenon is not a result of antagonism, even should we attribute antagonism to sensation and not to attention; it depends on the contrary upon a suppression of antagonism.

2nd. The sensations proceeding from the irritation of corresponding points are not identical to the extent of being indistinguishable, because then it would not be possible, by the aid of an instantaneous illumination, to distinguish between the relief of a stereoscopic image and the corresponding pseudoscopic effect.

3d. The fusion of two different sensations which correspond is not an effect of transient neutralization of one of these sensations, because the binocular impression of relief depends solely upon this, that we are simultaneously conscious of two different images. Now, this perception of relief is possible without displacement of the retinal image, and when the illumination endures only for an instant.

We are led to admit therefore that *two sensations, recognizeable the one from the other, arrive at our consciousness simultaneously without being fused;* that their fusion into one unique notion of the external object is therefore not done by a preestablished mechanism of the sensation, but by an act of consciousness.

4th. We find again that the corresponding impressions of the two retinas are similarly localised in the field of vision, or very nearly so, but that the representation which we make of one single object to which we refer the two impressions may sensibly derange this similarity. Now, if the similarity of localization was produced by an immediate act of the sensation it would not be possible that this sensation should be counter balanced by an opposing representation. It is otherwise if the similarity of the localization of the corresponding images rests upon the ocular evaluation, that is to say upon an evaluation of distances acquired by education or, in other terms upon the manner in which habit has taught us to interpret local signs. It is then only one experience which is contending with another; it becomes conceivable that the representation according to which two visual images appertain to the same object exerts an influence upon the estimation of their position by means of the ocular evaluation, and we understand that two distances a little different in reality may be estimated equal in the surface of the field of vision.

Finally, if the similar localization of corresponding points of the two visual fields does not rest upon a sensation, it must be that it is the same for the comparison of different distances in one field of vision. In fact, if the evaluation of the distances in the field of each of the eyes was given in sensation, there would necessarily result from it also, in immediate sensation, a complete concordance of both fields of vision, since there would be coincidence between both points of fixation and both meridians.

We see how the logic of facts impels us necessarily towards the empirical theory. I ought to say that in recent times efforts have been made to explain the perception of relief, and the phenomena of single and double binocular vision, by admitting a pre-arranged mechanism. In spite of all that is at the same time ingenious and elastic in the hypothesis upon which these theories are founded, attempts of this kind come into collision with an infinite variety of phenomena for which it is necessary to account, and all of which it is impossible for them to embrace. When a system of this kind adapt itself well to certain circumstances of vision, and seems to account for them, it is found that all the other cases remain unexplained. It is necessary then to have recourse to a very elastic hypothesis, in accordance with which in these other cases experience may annul a sensation. But what becomes of our perceptions if they are susceptible of being annulled by contrary representations? Since, in all cases, it is experience which decides as a last resort, to suppose that the notion is formed from the commencement as an effect of experience alone seems to me much more simple than to suppose the presence of sensations whose effects experience will have to combat in the majority of cases.

On the other hand, the divers systems of hypotheses which have erected to reconcile in succession the facts with the naturalistic theory are perfectly useless. We as yet know no single fact which is irreconcilable with the empirical theory; now, this presupposes no anatomical structure impossible to demonstrate, it does not demand of the nerves that they should act otherwise than they do every where else; it is founded solely upon the knowledge of the mechanism of the association of notions and of representations, a mechanism with which every day experience has made us sufficiently acqainted. It is true that a complete explanation of the psychical functions has not yet been given, and that it probably will not be given very soon. But, since these functions exist in fact and since no form of the naturalistic theory has been able to explain all without invoking them at times, every just spirit will have to avow that psychological facts may serve as a perfectly legitimate base for the theory of vision, although we have not as yet been able to construct a perfect theory for these same facts.

Among the notions which we have of the external world, it is impossible to place a boundary between that which is attributable to immediate sensation and that which is fonnded on experience. Whatever be the boundary line at which we stop, cases are still always found where experience takes on a eharacter of immediate precision and accuracy which put upon the second plane that which might be considered as an immediate result of sensation.

The empirical theory alone escapes all the contradiction of this kind. We have seen, in fact, that it considers all notions of space as resting on experience; it supposes that the local signs of our visual sensations, along with the qualities of these sensations, are nothing else than signs, the signification of which habit teaches us to interpret.

Now, the interpretation of these signs becomes easy to us only by observing the modifications which prove them viz: either our changes of position, or the movements which we give to external objects. The infant commences at first by playing with his hands; he does not yet know how to direct either his hands or his eyes towards the colored objects which attract his attention. Later he tries to take hold of objects, he turns and overturns them in every way, looks at them, tastes and feels of them on every side. He prefers the simplest objects; the most primitive playthings are more successful with him than the most refined inventions of modern industry. When, after having looked at his plaything frequently for some weeks, the child knows it in all its aspects, he throws it away and wants a new one. In this way he learns to know the different visual images furnished by the same object, in relation with the movements which his little hands can impress upon it. The corporeal representation thus obtained consists of the aggregation (*ensemble*) of all these visual images. When we have conceived an exact notion of the form of any object whatever, this is sufficient, in fact, to put our imagination in a condition to represent to us the impression which this object would give us by varying its position or our point of view. All these notions are included in the representation of the form of the object, and we can deduce them from it when we think of the movements which it would be necessary for us to make to obtain in reality these different aspects.

When we look at stereoscopic images we may often remark a phenomena well fitted to confirm what I have just said. It is often difficult to fuse immediately linear designs of complicated crystalline forms. I commence then by seeking in the first place for two corresponding points, and I try to fuse them by a voluntary movement of my eyes; but, so long as I have not apprehended the signification of the images, my eyes lose hold every instant, and the fusion is not maintained. But if I set myself to following with my sight the different lines of the figure, there comes a moment when I suddenly apprehend the corporeal form represented, and from that time my two visual lines run over the contours of the apparent body without the least difficulty, without ever again becoming disassociated. After the exact representation of the form of the body is manifested, there follows the reign of the movements of the eyes necessary for the vision of this body. To execute these movements is, so to speak, to translate our representations into the language of the real world; now, our representations being themselves a translation, we see that when, by our motions, we obtain the images which we expect, we make an experimental verification of the accuracy of our representation.

I think that what we have just seen is very important. The interpretation of our sensations rests upon experince, and not upon the simple observation of what passes externally. Experiment teaches us that the relation between two phenomena exists *at whatever moment* we may choose, and under conditions that we may change at our will. It teaches us the existence of a permanent relation of cause to effect, the constancy of this relation being demonstrated by this circumstance, that we can verify it at any minute. Even observation, repeated often under the most varied conditions, can scarcely attain such a degree of certainty. It teaches us, indeed, that the phenomena whose relation we study are often or always beheld together, but it does not prove that it must necessarily be always so with them. If we take, for example, those sciences of observation which have attained the highest degree of perfection, such as Astronomy, Meteorology or Geology, we find that the knowledge of the causes of phenomena attains a degree of perfect certainty, only when we have been able to make the same forces act experimentally in our laboratories. The non-experimental sciences have revealed to us, up to the present time, no new force; I think that here is a fact which is not without importance.

It is clear that habit enables us to arive at an interpretation of the local signs sufficient to deduce from them all the results susceptible of being verified by experience, that is to say, all the *real contents* of our notions. We have admitted, up to the present time, that the notions of space and of motion were obtained primarily by touch. It is evident that the only thing which we could learn at first was, that our voluntary motions produce modifications perceptible by sight and touch. The majority of these changes, which we can produce voluntarily, are only modifications in space, that is to say motions; we may certainly also by this means produce other changes inherent in the objects themselves. Are we able, without knowing it before hand, to recognize that the movements of our hands and eyes are indeed displacements of these organs, and not confound them with modifications of the objects themselves? I think so. The relations of space have this peculiarity that their modifications depend in no degree upon the nature of the body, while all other real relations between objects depend upon their qualities. It is as it regards vision that it is the easiest to assure ones self of this immediately. Whatever be the contents of the field of vision one self same motion of the eye, which produces a certain displacement of the retinal image, produces always the same series of changes; this motion causes the impressions which produced the local signs $a^0$, $a$, $a^1$, $a^2$, $a^3$ . . . ., to produce then the signs $b^0$, $b$, $b^1$, $b^2$, $b^3$, . . . . ; and this always takes place in the same way, whatever be the qualities of these impressions. This suffices to characterize these changes, to cause their peculiar nature to be recognized, and to assure us that they are changes in space. Experience, upon which we found our theory, finds here then a sufficient basis, and we have no need to consider the question of what, in the general notion of space, is given priori or a posteriori.

Some may consider he existence of illusions of the senses an objection to the empirical theory, and say that, if it is experience which has taught us to interpret our sensations, this interpretation ought always to be in accord with experience. We answer thus: When abnormal circumstances modify the retinal images, we continue to represent external objects to ourselves in such a manner as would give exact results in normal conditions, and this is what occasions the illusions in question. Now, in order that the manner of observing should be normal, it is not sufficient that the rays of light arrive without deviation as far as the cornea; it is still further necessary that the eyes should perform their functions in such a manner as to give the sharpest and most easily recognizable images. This demands that the different points of the contours of the object should be depicted successively upon the centres of the two retinas, and that we should cause our eyes to execute the motions which we call normal because they are the best fitted for the exact comparison of positions. When one of these conditions is departed from, illusions are produced. The longest known are those which are presented when the rays of light undergo a refraction or reflection before entering the eye. But the imperfect accommodation when we look through one or two little holes, an incorrect convergence during monocular vision, a displacement of the eyeball produced by a pressure of the finger, a muscular paralysis, &c., may all cause illusions relating to the position of objects. They may result also from an incorrect appreciation of certain elements of the sensation, such as the degree of convergence of the eyes, whose evaluation may easily be falsified by muscular fatigue. All these illusions are governed by this simple rule that *we always think that we have before us objects such as they must be to produce the same retinal images when the observation is normal.* If the images are of such a nature that similar ones could never be produced during normal observation, the judgment is governed by the normal observation which approaches it most nearly, and then we neglect most easily those elements of the sensation which are perceived with the least degree of certainty. When several interpretations are equally possible, we oscillate ordinarily from one to the other: but then it is possible to suppress this hesitation by striving to make as vivid a representation as possible of the image such as we wish to see it.

All this rests evidently upon what we may call false inductive conclusions. It is a question of reasonings where we do not consciously take account of analogous anterior observations, or do not weigh the influence which these facts ought to have upon the conclusion. It is for this reason that I have given them the name of *unconscious reasonings*; and if this denomination, accepted since by other partisans of the empirical theory, has shocked certain minds, it is because we have been in the habit of considering a reasoning as the highest manifestation of the conscious intelligence. Far from this, the reasonings which play so great a roll in our perceptions, can never be expressed under the logical form to which we are accustomed, and it is necessary to depart a little from the beaten roads of psychological analysis to assure ourselves that we have really to do here with an operation of the mind similar to those which we encounter in ordinary reasoning.

The difference between the reasonings of logicians and the inductive reasoning upon which rest the notions which the senses give us of the external world appears to me to be purely apparent, and seems to consist in this, that the first are susceptible of being expressed, which is not the case with the second, which, in place of words, are constituted only of sensations and memories of sensations. It is precisely in what it has to do with reasonings which words cannot express, that the difficulty resides which we experience in studying this whole chapter of the operations of the mind or even in speaking of them.

*(Concluded in our next number.)*

